# Correction: Predicting physician departure with machine learning on EHR use patterns: A longitudinal cohort from a large multi-specialty ambulatory practice

**DOI:** 10.1371/journal.pone.0315090

**Published:** 2024-12-03

**Authors:** Kevin Lopez, Huan Li, Hyung Paek, Brian Williams, Bidisha Nath, Edward R. Melnick, Andrew J Loza

There are errors in Table 2. The Value of Accuracy should have been 0.79 and the Metric FI should be Weighted F1. Please see the correct Table 2 here.

**Table 2:** Model performance at optimal threshold using Youden’s J index. **A** 2x2 confusion matrix for the optimal threshold showing physician-month counts and **B** classification performance statistics. PPV is Positive Predictive Value; NPV is Negative Predictive Value. Weighted F1 was computed as defined in the scikit-learn package (1.0.1).

**A**				**B**	
		**Predicted**		**Metric**	**Value**
		Retained	Departed	Sensitivity	0.64
**Ground Truth**	Retained	1523	397	Specificity	0.79
	Departed	17	30	PPV	0.07
				NPV	0.99
				Weighted F1	0.86
				Accuracy	0.79
