# Supplementary material for: Predicting physician departure with machine learning on EHR use patterns: A longitudinal cohort from a large multi-specialty ambulatory practice
Source: PLoS One. 2023 Feb 1;18(2):e0280251. doi: 10.1371/journal.pone.0280251 (PMC9891518; doi:10.1371/journal.pone.0280251)
Supplement: S1 Table — (DOCX) [file pone.0280251.s001.docx]

**S1 Table.** Feature list and variable types.

| **Feature Name** | **Feature Type** |
| --- | --- |
| age_group | Ordinal |
| gender | Categorical |
| calendar_month | Ordinal |
| covid_wave | Ordinal |
| patient_volume | Continuous |
| physician_demand | Continuous |
| physician_work_intensity | Continuous |
| panel_cnt | Continuous |
| risk_avg | Continuous |
| teamwork_on_inbox_value | Continuous |
| note_quality_manual_value | Continuous |
| note_quality_contribution_value | Continuous |
| number_of_rx_errors | Continuous |
| ehr_time_8 | Continuous |
| wow_time_8 | Continuous |
| note_time_8 | Continuous |
| order_time_8 | Continuous |
| ib_time_8 | Continuous |
| review_time_8 | Continuous |
| tenure | Ordinal |
| specialty | Categorical (1-hot encoded) |
| EWA_avg_patient_volume | Continuous |
| EWA_avg_physician_demand | Continuous |
| EWA_avg_physician_work_intensity | Continuous |
| EWA_avg_panel_cnt | Continuous |
| EWA_avg_risk_avg | Continuous |
| EWA_avg_teamwork_on_inbox_value | Continuous |
| EWA_avg_note_quality_manual_value | Continuous |
| EWA_avg_note_quality_contribution_value | Continuous |
| EWA_avg_number_of_rx_errors | Continuous |
| EWA_avg_ehr_time_8 | Continuous |
| EWA_avg_wow_time_8 | Continuous |
| EWA_avg_note_time_8 | Continuous |
| EWA_avg_order_time_8 | Continuous |
| EWA_avg_ib_time_8 | Continuous |
| EWA_avg_review_time_8 | Continuous |
| r_slope_patient_volume | Continuous |
| r_slope_physician_demand | Continuous |
| r_slope_physician_work_intensity | Continuous |
| r_slope_panel_cnt | Continuous |
| r_slope_risk_avg | Continuous |
| r_slope_teamwork_on_inbox_value | Continuous |
| r_slope_note_quality_manual_value | Continuous |
| r_slope_note_quality_contribution_value | Continuous |
| r_slope_ehr_time_8 | Continuous |
| r_slope_wow_time_8 | Continuous |
| r_slope_note_time_8 | Continuous |
| r_slope_order_time_8 | Continuous |
| r_slope_ib_time_8 | Continuous |
| r_slope_review_time_8 | Continuous |
