# Supplementary material for: Predicting physician departure with machine learning on EHR use patterns: A longitudinal cohort from a large multi-specialty ambulatory practice
Source: PLoS One. 2023 Feb 1;18(2):e0280251. doi: 10.1371/journal.pone.0280251 (PMC9891518; doi:10.1371/journal.pone.0280251)
Supplement: S2 Table — (DOCX) [file pone.0280251.s002.docx]

**S2 Table.** Model Comparison

| **Model** | **AUC** | **Sensitivity** | **Specificity** | **PPV** | **NPV** |
| --- | --- | --- | --- | --- | --- |
| Logistic Regression^a^ | 0.54 (0.47-0.63) | 0.30 | 0.82 | 0.04 | 0.98 |
| Gaussian Naïve Bayes^a^ | 0.60 (0.58-0.67) | 0.87 | 0.35 | 0.03 | 0.99 |
| Random Forest^a^ | 0.66 (0.56-0.71) | 0.51 | 0.74 | 0.05 | 0.98 |
| XGBoost | 0.82 (0.71-0.85) | 0.64 | 0.79 | 0.07 | 0.99 |

^a^ models required imputation of missing data.
