# Supplementary material for: Predicting physician departure with machine learning on EHR use patterns: A longitudinal cohort from a large multi-specialty ambulatory practice
Source: PLoS One. 2023 Feb 1;18(2):e0280251. doi: 10.1371/journal.pone.0280251 (PMC9891518; doi:10.1371/journal.pone.0280251)
Supplement: S3 Table — (DOCX) [file pone.0280251.s003.docx]

**S3 Table.** Demographics of physicians with missing tenure data.

| **Variable** | | **Present (n)** | **Missing (n)** | **p-value** |
| --- | --- | --- | --- | --- |
|  | |  |  |  |
| *Age Group* | |  |  | 0.055 |
|  | 25-34 | 10 | 0 |  |
|  | 35-44 | 66 | 0 |  |
|  | 45-54 | 94 | 1 |  |
|  | 55-64 | 79 | 3 |  |
|  | 55+ | 59 | 5 |  |
|  | |  |  |  |
| *Gender* | |  |  | 0.01* |
|  | Male | 178 | 9 |  |
|  | Female | 132 | 0 |  |
|  | |  |  |  |
| *Specialty* | |  |  | 0.97 |
|  | Internal Medicine | 156 | 4 |  |
|  | Family Medicine | 38 | 1 |  |
|  | Other specialty | 115 | 4 |  |

* denotes p-value <0.05
