# Supplementary material for: Predicting physician departure with machine learning on EHR use patterns: A longitudinal cohort from a large multi-specialty ambulatory practice
Source: PLoS One. 2023 Feb 1;18(2):e0280251. doi: 10.1371/journal.pone.0280251 (PMC9891518; doi:10.1371/journal.pone.0280251)
Supplement: S4 Table — (DOCX) [file pone.0280251.s004.docx]

**S4 Table.** Spearman correlation coefficients for interaction SHAP plots

|  | |  | | **Tenure** | | | | | | | | |
| --- | --- | --- | --- | --- | --- | --- | --- | --- | --- | --- | --- | --- |
| **Interacting Feature** | | **0-5** | **5-10** | | **10-15** | **15-20** | **20-25** | **25-30** | **30-35** | **35-40** | **40-45** | **>45** |
|  | EWA^a^ EHR time | -0.69* | -0.51* | | 0.18* | 0.02 | 0.09 | 0.62* | 0.79* | 0.05 | 0.38* | 0.18 |
|  | EWA inbasket time | -0.90* | -0.90* | | 0.85* | 0.84* | 0.82* | 0.74* | 0.57* | 0.83* | 0.18 | 0.72* |
|  | EWA order time | -0.62* | -0.47* | | 0.67* | 0.67* | 0.25* | 0.37* | 0.25 | 0.05 | -0.45* | -0.31 |
|  | EWA note time | 0.61* | -0.71* | | 0.39* | 0.56* | 0.69* | 0.10 | 0.19 | 0.35 | 0.49* | 0.46 |

* indicates p < 0.005 (Bonferroni corrected value equivalent to 0.05 for single test)

^a^ EWA is exponential weighted average
