# Supplementary material for: Predicting physician departure with machine learning on EHR use patterns: A longitudinal cohort from a large multi-specialty ambulatory practice
Source: PLoS One. 2023 Feb 1;18(2):e0280251. doi: 10.1371/journal.pone.0280251 (PMC9891518; doi:10.1371/journal.pone.0280251)
Supplement: S1 Fig — Permutation importance sampling was performed to test the global significance of each feature in model predictions. Four features showed statistical significance with p < 0.05: Tenure, Exponential Weighted panel complexity, Exponential weighted teamwork on inbox, and rolling slope of the panel count. (DOCX) [file pone.0280251.s005.docx]

**S1 Figure**. Permutation importance sampling. Permutation importance sampling was performed to test the global significance of each feature in model predictions. Four features showed statistical significance with p < 0.05: Tenure, Exponential Weighted panel complexity, Exponential weighted teamwork on inbox, and rolling slope of the panel count.
