# Supplementary material for: Predicting physician departure with machine learning on EHR use patterns: A longitudinal cohort from a large multi-specialty ambulatory practice
Source: PLoS One. 2023 Feb 1;18(2):e0280251. doi: 10.1371/journal.pone.0280251 (PMC9891518; doi:10.1371/journal.pone.0280251)
Supplement: Attachment — Submitted filename: Response to Reviewers.docx [file pone.0280251.s006.docx]

**Response to Reviewers**

In the revised version of this manuscript, we have responded to the reviewers’ comments and suggestions. In addition to correcting textual errors and providing clarifications on topics, we have added multiple substantive analyses to the manuscript. First, a new Table 1 summarizing the physician demographic and EHR use characteristics stratified by physician departure status has been added as part of the additional exploratory data analyses asked for by Reviewer 2. We have also compared the performance of our primary model to three additional machine learning methods for prediction: logistic regression, naïve bayes, and random forests. The comparison shows the performance advantage of the chosen primary model, and these analyses are summarized in a new supplementary table. We have also performed multiple new analyses to inform the interpretation of features driving prediction. To assess global significance of features, we performed permutation importance sampling. We have also provided quantification of the nonlinearity and associations present in the Shapley plots to support the descriptions in the text with statistical tests as suggested by Reviewer 1. We thank the reviewers for these suggestions and believe that these changes have led to an improved manuscript.

**Reviewer 1**

1. Not sure what “tenure” means in this non-teaching, university-based practice in the abstract. Maybe use “time since hiring” at this time, or define in the abstract

Tenure was chosen for this term due to brevity and its use in other fields to denote the duration of time in a given position at a company. We appreciate that this could be better defined due to the most common use of the word “tenure” in academia. We have added definitions of the term to the abstract and the methods section. These changes read:

*…tenure (time since hiring date)...*

2. P3. “Alternatively, EHR audit log data have shown tremendous potential in tracking how physicians” - no need for “alternatively” as not related to prior paragraph.

This extra word has been removed.

3. P1. “All ambulatory physicians practicing in the network were eligible.” - In the abstract it noted that teaching clinicians were excluded? Why only cardiologists?

The practice network where this study was conducted consists of non-teaching clinicians only, so this descriptor was included to provide information on the physicians in the study rather than a selection criterion that was imposed.

A total of 25 subspecialties were present among physicians in the study. We clarified the writing to show this. The three specialties with the largest representation are now enumerated, and the remainder are mentioned to help clarify this issue.

*A wide range of specialties were represented in the sample including 119 (37.3%) internal medicine, 39 (12.2%) family medicine, 36 (11.3%) cardiology, and 22 additional specialties with 5% or fewer physicians.*

4. No comment of EHR Go-live date. If this was different for different clinicians/sites, it should have been included, if all the same, would mention this in the methods.

All sites had an EHR Go-live date of 5 or more years prior to the start of the study. This has been added to the methods.

5. Data sources/variables. “Remained employed by the practice network in the broad categories of physician characteristics, HER use, clinical productivity, and temporal trends and events” -Do not understand this sentence. I would not connect employment to the categories listed. Could this be clarified?

The phrase “while employed” was included to denote that data prior to the listed hire date or after the listed departure date was not used in this study. This may arise from discrepancies in exact dates between Human Resources and EHR records. This wording has been changed to make this more clear.

*Monthly observations were collected for each physician participant throughout the study period in the broad categories of physician characteristics, EHR use, clinical productivity, and temporal trends and events. Any data on EHR use prior to the hire date and subsequent to the departure date was not included.*

6. Data sources/variables. “Reasons for departure included leaving the practice network for an outside position, leaving medicine, and retirement.” - How was this determined? Not something that would be mandatory non-research related? Were the departing clinicians asked? Were they consented?

Reasons for departure for specific individuals were not known as this data is not collected by the HR department, but involuntary departures were excluded. The methods section has been updated.

*Physicians who were involuntarily terminated were excluded from the analysis. Specific voluntary reasons for departure were not available.*

7. Data sources/variables. “Number of prescription errors (total of wrong medication, wrong patient, and wrong formulation retract and reorder errors). (41) -This reference only measured wrong patient errors. How were wrong medication and wrong formulation errors determined? I think that this is an error and only wrong patient errors were measured if the method from ref. 41 was used.

We thank the reviewer for identifying this. Clarifying this data source shows that the prescription error variable included in this model measures likely wrong patient errors based on the retract and reorder model used in reference 41. The text has been edited as follows to correct this.

*… and number of prescription errors (total retract and reorder errors).*[*(41)*](https://paperpile.com/c/BQIpaq/Lx88)

8. Data sources/variables. “Panel count (primary care only, number of patients seen in the prior two years),” -First, I assume that the intent is the number of UNIQUE patients seen in the prior two years. Second, is this really a measure of panel size? A clinician seeing only acute complaints could have a high panel count despite not provide primary care. In addition, if using this definition, the sum of the panel sizes of all the PCP’s can be significantly larger that the panel size of the entire clinic. Typically, only one clinician is assigned to each patient. I am OK with the variable as it is clear, but not sure that it means or should be called panel size. Maybe call it “unique patients seen”.

Clarification has been made to the definition used for Panel Count. This does refer to the number of unique patients seen in the prior two years and for whom the provider is designated as Primary Care Provider within the EHR.

*… panel count (number of unique patients seen in the prior two years for whom the physician was listed as Primary Care Physician in the EHR; this variable was set to missing for providers who were not in the specialties Internal Medicine, Family Medicine, or Pediatrics), …*

9. There is no discussion on different sites. Did all the clinicians come from the same geographic site? If so, would mention this. If not, why wasn’t site a variable?

Physicians did not come from the same site, however this variable had to be removed as part of de-identifying the data. We realize this is a limitation of the data as the site may have specific effects on both EHR use characteristics and departure rates. This has been added as a limitation in the discussion section.

*There are additional variables which were not available for the analysis that may be important features in prediction of departure. One of these is practice location as certain sites may have different EHR use patterns or physician turnover rates. Practice location was not available as part of the deidentification process.*

10. Performance on the unseen testing dataset yielded an ROC Area Under the Curve (AUC) of 0.80 - This study was somewhat underpowered as only 44 clinicians departed. As such, it would be nice to see the CI of the AUC to get an idea how much uncertainty the somewhat small sample is producing.

The confidence interval on the AUC as well as a confidence band on the ROC curve have been added to help interpret the precision of these study results. The ROC-AUC 95% CI is 0.71 - 0.85. We have also included the Precision-Recall Curve and its AUC as a second panel in Figure 1 as this may be more directly of interest to readers given the low frequency of departure events.

11. F1 is not defined

The definition has been added to the legend of new Table 2 (Formerly Table 1).

*F1 is the harmonic mean of PPV and Sensitivity.*

12. While 3a is specifically discussed, it in not clearly stated where 3b, 3c and 3d are being talking about.

References to the subpanels 3B-D have been added to appropriate areas in the results text.

*Panel complexity also showed a nonlinear contribution with peak risk contribution at approximately 1.7 (****Figure 3B****). The lowest and highest age groups had the highest risk of departure with protective effects estimated for most individuals aged 35 to 54 (****Figure 3C****). Interactions are evident, especially in these protective age groups. High exponentially weighted average documentation time diminished the estimated protective effect for physicians in these age groups. Low physician demand was associated with increased risk of departure, and risk decreased nonlinearly at high demand, however variation in panel count for a given level of physician demand was not readily apparent as would be present in an interaction (****Figure 3D****).*

13. From a statistical point of view, this text talks about non-linear changes in the risk of departure, such as high then down, then back up for instance. The outcome only had 44 events. Does this data set have the ability to statistically find these non-linear changes in the in all these variables. It is claimed in Figure 2 that these are 67 features. Is there really enough power to suggest that these non-linear relationships with the outcome are significantly better than linear, or just noise?

This is an excellent point and we have assessed both the trend linearity and associations between SHAP values and interaction values that are discussed in the text using statistical methods. We agree with the reviewer that this provides more solid context for the relationships that were qualitatively described. For linearity descriptions, we have performed the linear RESET test which compares a base linear model with one containing higher order terms to determine if there is significant nonlinearity. For descriptions regarding associations between SHAP values for a primary feature at a given value and a secondary interacting feature, we have computed Spearman Rank Correlation coefficients to provide the reader with the strength and significance of these correlations. The text in the methods and results sections have been updated.

*Relationships between the SHAP values and feature values were assessed for nonlinearity with the linear RESET test (python package statsmodels 0.14.0). Spearman correlation was computed to examine associations between SHAP values for a primary feature and the value of interacting features (python package scipy 1.7.3).*

*…*

*Feature dependence plots for the top four features from the SHAP beeswarm plot in Figure 2 are displayed in* ***Figure 3*** *to show the relationship between feature value and SHAP value, as well as possible interactions between features. Tenure contributed nonlinearly to departure risk (linear RESET test p < 0.001), with highest risk at low tenures and initially decreasing before rising again at higher tenures (****Figure 3A****). The vertical dispersion indicates that interactions are present. The feature with the greatest estimated interaction was the exponentially weighted average EHR time normalized to an 8-hour day. Within ordinal tenure groups by decade, high EHR times have different contributions. High EHR times tended to decrease risk of departure for physicians with tenure from 0-5 and 5-10 years (spearman correlation -0.43 and -0.50, respectively with p < 0.001), but were associated with higher risk of departure for longer tenures (spearman correlations ­­0.43, 0.46, 0.4, 0.28, 0.64 for 10-35 years, all p < 0.001; spearman correlations 0.17 p = 0.38, 0.02 p = 0.92, 0.40 p = 0.003, -0.31 p = 0.22). Panel complexity also showed a nonlinear contribution (linear RESET test p < 0.001) with peak risk contribution at approximately 1.7 (****Figure 3B****). The lowest and highest age groups had the highest risk of departure with protective effects estimated for most individuals aged 45 to 64 (****Figure 3C****). Interactions are evident, especially in these protective age groups. High exponentially weighted average documentation time was associated with higher SHAP values for physicians in these age groups (spearman correlation coefficient between SHAP value and interaction value 0.59 and 0.52 respectively with p < 0.001). Low physician demand was associated with increased risk of departure, and risk decreased nonlinearly at high demand (linear RESET test p < 0.001), however variation in panel count for a given level of physician demand was not readily apparent as would be present in an interaction (****Figure 3D****).*

Supplemental Table 4 has been added collating the spearman correlations coefficients supporting the description of trends in Figure 4 and is referenced in the text.

*A complete list of spearman correlation coefficients quantifying associations between interaction SHAP values and the interaction feature value is available in* ***Supplemental*** ***Table 4****.*

Regarding the performance of classifiers with nonlinear components, additionally model comparisons have been made (see Supplemental Table 2) and demonstrate significant improvement over linear models (logistic regression in this table).

***Supplemental Table 2.*** *Model Comparison*

| ***Model*** | ***AUC*** | ***Sensitivity*** | ***Specificity*** | ***PPV*** | ***NPV*** |
| --- | --- | --- | --- | --- | --- |
| *Logistic Regression^a^* | *0.54 (0.47-0.63)* | *0.30* | *0.82* | *0.04* | *0.98* |
| *Gaussian Naïve Bayes^a^* | *0.60 (0.58-0.67)* | *0.87* | *0.35* | *0.03* | *0.99* |
| *Random Forest^a^* | *0.66 (0.56-0.71)* | *0.51* | *0.74* | *0.05* | *0.98* |
| *XGBoost* | *0.82 (0.71-0.85)* | *0.64* | *0.79* | *0.07* | *0.99* |

*^a^ models required imputation of missing data.*

14. There was also no discussion about which variables were actually statistically significantly associated with the outcome? If decision tree analysis with Gradient Boost is unable to test if a feature is statistically associated with an outcome, this needs to be explained in the methods or results. If this method cannot produce statistically analysis, would consider running a secondary logistic regression to at least show which features are statistically associated with the outcome

The ROC difference from the dashed diagonal line (allowing for the uncertainty shown in the confidence band now displayed in the ROC curve) shows significance of the overall fit over a random classifier. Determining the statistical significance of individual features in this model can be assessed by the estimated total feature importance through permutation importance sampling. This method permutes feature values to compare the change in ROC-AUC with random feature values versus the actual values in the data set. This permutation importance sampling analysis has been added as supplemental Figure S1 and referenced in the methods and results sections.

15. In results page 5 (paragraph 3) says ages 35-54 are protective from departure when in discussion page 7 (paragraph 2) says ages 45-64 are protective which is supported by Figure 3c.

We thank the reviewer for identifying this error, it has been fixed.

16. Figures 4 has 2 panels that are labelled “Tenure and Exp. weighted EHR time”?

This error has been fixed.

17. P1: “The highest contribution of tenure to departure risk came for physicians whose tenure data was missing.” - This is a limitation of the data. If the method of using this missingness as a predictor were to be recommended, it would not be useful as any missing tenures could be found. What would be more interesting would be to try to figure out if the missingness had a bias? Maybe older physicians? Younger?

We agree that this feature value serves as a marker for a confounding variable and if this method were to be generalized to a different practice where records were entirely complete, this finding would likely not apply. We view this as a specific instance of the underlying problem facing generalization of prediction methods. It is likely that to achieve similar performance in a different medical practice, this algorithm would have to be tuned for local use. This limitation has been added to more extensive discussion points.

Regarding the type of physicians who had missing tenure data, secondary analysis shows a trend towards older age (p = 0.055), more male (p = 0.01), and no significant association with tenure. This analysis has been compiled and added as Supplemental Table 3. The results section has been updated as follows:

*Physicians with missing tenure data were more likely to be male with a trend towards older age groups (Supplemental Table 3).*

**Reviewer 2**

1. Introduction section needs to be re-written to improve its quality and readability.

We have carefully reviewed the introduction for syntactical issues and copy edits and made changes accordingly to improve readability. If there are specific sentences or paragraphs with concern for quality, we are happy to address further.

2. What is the motivation of the proposed work? Research gaps, objectives of the proposed work should be clearly justified

We have edited the statement summarizing the objective of the proposed work in the introduction.

*In the present study, we extend the cohort through the Covid-19 pandemic and develop a more comprehensive predictive model by adding multiple new metrics more recently found to be associated with burnout and using machine learning techniques to identify early predictors of physician departure with internal validation on an unseen hold-out data set.*

3. Summarize the dataset used in table (Features, Continuous/Categorical, Sample values)

This has been added as Supplemental Table 1.

4. The literature has to be strongly updated with some relevant and recent papers focused on the fields dealt with in the manuscript.

We have reviewed the literature and believe that the key papers have received citations, however, if there are specific works that we are missing, we would gladly add them.

5. Authors used only XGBoost while there are several Machine learning algorithms such as SVM, KNN, GBM, LR, Random forest. Authors need to compare the results with these state of art algorithms

We thank the reviewer for the suggestion to compare to other machine learning algorithms. We compared performance of the model used in the manuscript with logistic regression, Naive Bayes, Random Forest Classifier. The results have been added as Supplemental Table 2 and referenced in the Results section.

*Performance of the XGBoost classification algorithm in predicting departure on the unseen testing dataset yielded an ROC Area Under the Curve (AUC) of 0.80 and an accuracy of 97% as shown in* ***Figure 1****. The optimal threshold for model performance determined by Youden’s J index occurred with a log-odds threshold of 0.002 with sensitivity was 0.64 and specificity was 0.79. Additional classification statistics for this threshold are shown in* ***Table 1****. Performance of this model exceeded that achieved by logistic regression, naïve Bayes, and random forest classifiers (****Supplemental*** ***Table 2****).*

6. Explain why the current method was selected for the study, its importance and compare with traditional methods.

Performance of this model compared to other methods has been added to the results. Additional discussion has been added on the strengths of the method and improved performance over those tested in point 5. These points have been made in conjunction with response to point 7.

*Performance of this model exceeded that achieved by logistic regression, naïve Bayes, and random forest classifiers (****Supplemental*** ***Table 2****). Performance of this model exceeded that achieved by logistic regression, naïve Bayes, and random forest classifiers (****Supplemental*** ***Table 2****) leading us to choose the XGBoost model as the primary predictive tool for the study.*

*Machine learning models provide a flexible approach to tasks where a set of important features is hypothesized, but the direct relationships between these features and the outcome is not known and may include non-linearities and interactions. The ability to detect complex interactions is a strength of the model presented here and expands current knowledge of factors associated with physician departure.*[*(7,22,47)*](https://paperpile.com/c/BQIpaq/bI88+FGdt+KtHiO)*. Among the machine learning classification algorithms tested in this study, XGBoost provided the most accurate performance and did not require imputation of missing data. These are two strengths which led to its choice as the primary predictive model for the study.*

7. Authors are suggested to include more discussion on the results and also include some explanation regarding the justification to support why the proposed method is better in comparison towards other methods

Additional discussion on the advantages of the main method have been added. This discussion has been enhanced by the addition of the analyses suggested in point 5. These points have been made in conjunction with response to point 6.

*Performance of this model exceeded that achieved by logistic regression, naïve Bayes, and random forest classifiers (****Supplemental*** ***Table 2****). Performance of this model exceeded that achieved by logistic regression, naïve Bayes, and random forest classifiers (****Supplemental*** ***Table 2****) leading us to choose the XGBoost model as the primary predictive tool for the study.*

*Machine learning models provide a flexible approach to tasks where a set of important features is hypothesized, but the direct relationships between these features and the outcome is not known and may include non-linearities and interactions. The ability to detect complex interactions is a strength of the model presented here and expands current knowledge of factors associated with physician departure.*[*(7,22,47)*](https://paperpile.com/c/BQIpaq/bI88+FGdt+KtHiO)*. Among the machine learning classification algorithms tested in this study, XGBoost provided the most accurate performance and did not require imputation of missing data. These are two strengths which led to its choice as the primary predictive model for the study.*

8. Does this kind of study have never attempted before? Justify this statement and give an appropriate explanation to do so in this paper.

To our knowledge the prior study conducted by this group is the only study examining EHR use features and practice departure. However, this model is set apart by the addition of new features, the choice of analytical methods, the longer study period through the pandemic, and the focus on prediction and validation in a hold-out data set rather than just parameter inference. A statement regarding this has been clarified in the Introduction

*In the present study, we extend this cohort through the Covid-19 pandemic and develop a more comprehensive predictive model by adding multiple new metrics more recently found to be associated with burnout and using machine learning techniques to identify early predictors of physician departure with internal validation on an unseen hold-out data set.*

9. Quality of figures is so important too. Please provide some high-resolution figures. Some figures have a poor resolution.

The editorial manager platform includes our high resolution images that the reviewer can download by clicking on the link at the top of each image in the compiled PDF. To avoid this being an issue with the revision, we have improved the figure quality and also included high resolution images in the Word document. Vector graphics figures have been provided where possible.

10. Add Exploratory data analysis section discussing the dataset.

To address this, we have added a new Table 1 which summarizes key feature values at the physician level between those who departed during the study and those who were retained. The results have been updated as well.

*The cohort included 319 physicians from 26 medical specialties over a period of 34 months producing a total of 9928 physician month observations which were all included in the analysis. Among the 319 physicians, 132 (41.4.%) were women and 95 (29.7%) were aged 45 to 54 years. A wide range of specialties were represented in the sample including 119 (37.3%) internal medicine, 39 (12.2%) family medicine, 36 (11.3%) cardiology, and 22 additional specialties with 5% or fewer physicians. A total of 44 (13.8%) physicians departed during the study period. In comparison of physician demographic features for those who departed during the study and those who were retained, there was a significant difference across age groups (p < 0.001) with higher departure rates occurring in the 35-44 and ≥ 65 age range, there was no significant difference in gender (p = 0.2), and there was no significant difference across specialties.*

11. Authors mentioned "Several variables significantly contributed to predicting physician departure". How authors concluded that?. Provide valid proof for this claim. Provide results with and without these variables.

We thank the reviewer for noticing this. To quantify the significance of features, we have now performed permutation importance sampling and added this as a supplemental figure, Figure S1. This method shows that 4 features have statistically significant global permutation importance with p < 0.05: Tenure, Exponential Weighted panel complexity, Exponential weighted teamwork on inbox, and rolling slope of the panel count.

*Figure S1. Permutation importance sampling. Permutation importance sampling was performed to test the global significance of each feature in model predictions. Four features showed statistical significance with p < 0.05: Tenure, Exponential Weighted panel complexity, Exponential weighted teamwork on inbox, and rolling slope of the panel count.*

12. What kind of hyperaparameter selection strategy is used?

Hyperparameters were selected using the grid-search cross-validation method from the scikitlearn python package. Once hyperparameters were selected. This has been emphasized in the methods for clarity:

*Hyperparameter tuning was accomplished using 5-fold grid-search cross-validation (python package sklearn version 1.0.1) on the training set for tree depth and L2 regularization weight.*

13. Overall writing, results and presentation needs improvement

Through the revision process, multiple edits and rephrasing have been performed for clarity. We are happy to make further specific changes as directed.

14. Try using feature selection algorithms and feature importance representation.

Feature importance computations have been now performed as discussed in point 11.
